# Increased maternal exercise of moderate intensity improves pregnancy outcomes of gestational diabetes mellitus patients through maintaining the balance of the gut microbiota

**DOI:** 10.3389/fmicb.2025.1526714

**Published:** 2025-05-29

**Authors:** Mengyan Xu, Xinru Ye, Fengcheng Cai, Yingying Wu

**Affiliations:** ^1^Nursing Department, Hangzhou Women’s Hospital (Hangzhou Maternity and Child Health Care Hospital), Hangzhou, China; ^2^The First Clinical Medical College, Zhejiang Chinese Medical University, Hangzhou, China

**Keywords:** gestational diabetes mellitus, gut microbiota, exercise, biodiversity, *Faecalibacterium*

## Abstract

**Background:**

Exercise therapy can reduce insulin resistance during pregnancy and improve glucose tolerance in women with gestational diabetes mellitus (GDM), leading to better pregnancy outcomes. This study aimed to investigate the effects of different exercise levels on GDM from the perspective of gut microbiota.

**Methods:**

Ninety patients with GDM were enrolled and divided into two groups: the L (*n* = 50) and the M (*n* = 40) groups. The L group performed 150 min of aerobic exercise per week, while the M group exercised for 200 min per week. After 8 weeks of intervention, fecal samples from each subject were collected for 16S rRNA gene sequencing.

**Results:**

Different exercise levels significantly affected membrane rupture and gestational weight gain in GDM patients (*p* < 0.05), but these effects were not significantly correlated by logistic regression analysis (*p* > 0.05). After sequencing, 4,712 OTUs and 3,483 OTUs were identified in the M and L groups, respectively, with 2,643 OTUs overlapping between both groups. Compared to the L group, the α-diversity in the M group was significantly increased (*p* < 0.05). The dominant phyla were *Firmicutes*, *Actinobacteriota*, and *Bacteroidota*. Compared to the L group, the M group had a significantly higher abundance of *Firmicutes* and a significantly lower abundance of *Actinobacteriota*. At the genus level, LEfSe analysis revealed that moderate-intensity exercise increased the levels of *Faecalibacterium*, *Agathobacter*, *Roseburia*, and *Osillospira*, but decreased the abundance of *Bifidobacterium* and *Coprobacillus*.

**Conclusion:**

There were significant differences in the composition and structure of the gut microbiota of patients with GDM with different exercise levels.

## Introduction

Gestational diabetes mellitus (GDM) is defined as the onset or initial discovery of abnormal glucose tolerance during pregnancy (Szmuilowicz et al., 2019). Glucose reaches the fetus through the placenta and increases fetal insulin production, which in turn stimulates fetal growth, resulting in macrosomia and larger-for-gestational-age babies (Sweeting et al., 2022). In the short term, GDM is associated with an increased risk of adverse pregnancy outcomes such as premature delivery, cesarean section, preeclampsia, fetal abnormalities, and intrauterine death (Clarke et al., 2020; Lee et al., 2017). There is also a long-term risk of maternal and offspring childhood obesity and type 2 diabetes (Kurtzhals et al., 2018), which seriously affects the quality of the birth population and public health. According to the epidemiological survey statistics from the International Diabetes Federation, the global incidence of GDM among pregnant women aged 20–49 years is as high as 14%, reaching 21.8% in some areas of China (Spaight et al., 2016; Dłuski et al., 2022). These findings indicate that approximately 1 in 5–6 pregnant women is diagnosed with GDM (Plows et al., 2018). In recent years, this trend has increased significantly due to the promotion of overnutrition and sedentary lifestyles resulting from China’s rapid economic development (Juan and Yang, 2020). Therefore, the need for treatment is increasing.

The cornerstone of GDM management is blood glucose control, and exercise has been shown to improve blood glucose control in patients with GDM (Alfadhli, 2015). According to a population-based cross-sectional study in Tianjin, China, increased physical activity during pregnancy was associated with a reduced risk of GDM among Chinese pregnant women (OR = 0.81, 95% CI: 0.67–0.97) (Leng et al., 2016). A systematic review and meta-analysis of longitudinal or cohort studies involving 30,871 pregnant women reported that, compared to no physical activity, any physical activity before or during the first trimester of pregnancy was associated with a 30 and 21% reduction in the odds of GDM, respectively. Furthermore, having more than 90 min of leisure-time physical activity per week before pregnancy was associated with a 46% lower risk of GDM (Mijatovic-Vukas et al., 2018). These findings indicate that exercise therapy, as a simple, economical, and effective noninvasive treatment, can reduce baseline insulin resistance (IR) during pregnancy and improve glucose tolerance in patients with GDM, thereby leading to better pregnancy outcomes (Shepherd et al., 2017; Xie et al., 2022). However, the specific mechanisms by which exercise impacts GDM remain unclear. In particular, there is a serious lack of studies on the regulatory effects of different levels of exercise on body diseases. As a result, the precise intervention of exercise therapy in GDM management still faces significant challenges.

In recent years, an increasing number of scientific studies have shown that intestinal microecosystems are closely related to the regression of various obstetrical and gynecological diseases (Kaisanlahti et al., 2023; Ribeiro et al., 2023). A previous multiomics analysis demonstrated that insufficient circulating dopamine, unbalanced short-chain fatty acid production, and excessive metabolic inflammation driven by the gut microbiome were closely associated with the development of GDM (Ye et al., 2023). Therefore, there is a relationship between gut microbiota and GDM, and gut microbiota may serve as a biomarker for the early detection of GDM. It could also be considered a potential modification target to reduce the risk of GDM (Medici Dualib et al., 2021). Additionally, exercise, as a potential environmental stimulus, has been reported to influence the body’s metabolic function and immune regulation by affecting the composition and structure of the gut microbiota (Cronin et al., 2018). Earlier evidence has shown that more than 2,500 species of gut microbiota differ between exercised and hypoactive mice (Choi et al., 2013). Increasing scientific evidence indicates that varying levels of exercise lead to distinct changes in gut microbiota. It was demonstrated that the abundance of gut microbiota in free rotation exercise mice (low-intensity exercise) and compulsory treadmill exercise mice (high-intensity exercise) differed significantly after 48 h of exercise (Allen et al., 1985; Pan et al., 2019). Another study found that athletes who exercised for more than 11 h per week had a significantly higher proportion of ≥ 2.5% *Prevotella* compared to low-exercise athletes (Petersen et al., 2017; Evans et al., 2014). Taken together, these findings suggest that different exercise levels not only change the abundance of gut microbiota but also affect the levels of specific intestinal species.

However, the effects of different exercise levels on the gut microbiota of patients remain unknown. In this study, we hypothesized that different exercise levels influence the progression of patients with GMD by regulating the gut microbiota. Based on this assumption, patients with GDM were enrolled and assigned to perform either low-duration exercise with moderate intensity (L group) and high-duration exercise with moderate intensity (M group) to investigate the effects of different exercise levels on GDM improvement. Subsequently, fecal samples from the GDM patients treated with the two exercise levels were collected, and 16S ribosomal RNA (16S rRNA) gene sequencing was employed to assess the regulatory effect of exercise therapy on GDM from the perspective of gut microbiota. The study also aimed to determine the influence of different exercise levels on the clinical characteristic and composition of gut microbiota in GDM patients.

## Methods

### Study design

From May 2021 to January 2023, 90 patients diagnosed with GDM who received daily antenatal care and delivery at Hangzhou Maternity and Child Health Care Hospital (Hangzhou, China) voluntarily enrolled in this study, meeting the inclusion and exclusion criteria. The diagnosis of GDM was based on the criteria published by the International Association of Diabetes in Pregnancy Specialist Groups (IADPSG) in 2010 (Metzger et al., 2010). The inclusion criteria were as follows: primary labor patients aged 20–35 years diagnosed with GDM between 24 and 28 weeks of pregnancy, with a single fetus, cephalic fetal presentation, no severe obstetric or surgical complications, and adequate language reading and writing comprehension skills. The exclusion criteria were as follows: patients with a family history of diabetes mellitus or diabetes mellitus combined with pregnancy; patients exhibiting symptoms for which international exercise guidelines prohibit physical activity during pregnancy, or who were advised by their doctors not to exercise for other reasons; patients with serious dietary restrictions or who had used antibiotics, probiotics, and insulin before or during pregnancy; and those unable to cooperate with the researcher due to mental disorders.

Additionally, epidemiological investigations were conducted for all participants, including general information, personal maternity history, living habits (diet, exercise, etc.), family medical history, and clinical data (such as antenatal examinations and nutrition consultations). This study was conducted in accordance with the Declaration of Helsinki and was approved by the Ethics Committee of the Hangzhou Maternity and Child Health Care Hospital (approval no. [2021] YilunshenA (1)-3). All participants were informed of their right to withdraw from the study at any time, and each provided signed, written informed consent.

### Patient grouping and sample collection

The enrolled participants were randomly divided into two groups: low exercise (L, *n* = 50) and moderate exercise (M, *n* = 40) group. The subjects in the L group performed 150 min of aerobic exercise per week for 8 weeks, whereas the participants in the M group exercised for 200 min per week for 8 weeks. In this study, we adopted a diversified approach to aerobic exercise, allowing patients to choose one or multiple forms of exercise daily to meet their exercise requirements. These forms include, but are not limited to, walking, cycling, stair climbing, prenatal exercises, prenatal yoga, and birth dance. The exercise intensity was set at moderate or higher, with a frequency of no less than five sessions per week. The daily cumulative exercise duration was at least 30 min, and the total weekly moderate-intensity exercise volume was no less than 150 min (L group) and 200 min (M group) (Wu et al., 2022). The exercise volume was quantitatively monitored using the UK-based Axivity Monitors (AX3) activity tracker. The AX3 is a triaxial accelerometer and optical sensor capable of sampling at frequencies between 12.5 and 3,200 Hz, and it is certified for use in pregnant women. Before exercise, the AX3 was configured according to the standard operating procedures to record exercise data, and it was worn on the participant’s wrist. This device accurately and conveniently recorded 24-h moderate-intensity exercise volume weekly. The data could be imported via USB for retrospective review and prospective evaluation in the backend system.

At the designated time points, maternal external signs such as height, weight, blood pressure, uterine height, and abdominal circumference were measured using tools including a soft tape measure, ultrasonic weighing scale, and electronic blood pressure monitor. Internal laboratory indicators, including glucose levels (fasting blood glucose, FBS; 2-h postprandial glucose, 2hPG) and lipid profiles (total cholesterol, TC; triglycerides, TG; high-density lipoprotein cholesterol, HDL-C), were assessed using the hexokinase method (G-6-PDH), glucose dehydrogenase method (blood glucose test strips), and a fully automated biochemical analyzer (Hitachi 7,600). All blood sample analyses were conducted as part of routine prenatal examinations to avoid additional sample collection from the patients.

Additionally, 30 g of fresh feces were collected from all participants. The fecal samples were immediately placed in a sterile anaerobic reservoirs, labeled sequentially, and stored in a-80°C ultra-low temperature freezer for subsequent 16S rRNA gene sequencing.

### 16S rRNA gene sequencing

After 8 weeks of exercise intervention (about 32–36 weeks), fecal samples were sent to Shanghai Majorbio Co., Ltd. (Shanghai, China) for 16S rRNA gene sequencing. Briefly, the total genomic DNA of fecal samples was isolated using the QIAamp DNA isolation kit (Qiagen, Hilden, Germany) according to the manufacturer’s instructions. The concentration and integrity of the isolated total DNA were evaluated using ultraviolet spectrophotometry and 1% agarose gel electrophoresis, respectively. The DNA samples were then amplified in the V3-V4 region of the 16S rRNA gene using barcoded primers (F: ACTCCTACGGGAGGCAGCA; R: GGACTACHVGGGTWTCTAAT), and the PCR products were recovered and purified using 2.0% agarose gel electrophoresis and gel recovery reagent from AXYGEN. The recovered products were quantified using the Quant-iT PicoGreen dsDNA Assay Kit and mixed at equal concentrations. Sequencing libraries were prepared using the TruSeq Nano DNA LT Library Prep Kit for the same amount of DNA and the quality of the sequencing libraries was evaluated using an Agilent High Sensitivity DNA Kit. Quantification of the established sequencing libraries was performed using the Quant-iT PicoGreen dsDNA Assay Kit and Promega QuantiFluor system, according to the manufacturer’s protocols. Finally, 16S rRNA gene sequencing was performed using a MiSeq sequencer with the MiSeq Reagent Kit V3 (600 cycles). The 16S rRNA sequencing data have been deposited in the Genome Sequence Archive (GSA)^1^ at accession number CRA010697.

### Sequencing data analysis

Raw sequencing data were analyzed using QIIME2 software (version 2019.4). The raw data were filtered and assembled with high quality, and chimeric sequences were removed using the DATA2 method to generate unique amplicon sequence variants (ASVs). Subsequently, the Greengenes reference database classifier (version 13.8) was utilized to annotate operational taxonomic units (OUTs) at a 97% similarity level based on the classify-sklearn algorithm. The diversity indices, including α-diversity (Shannon, Simpson, and Chao) and β-diversity (Principal Coordinate Analysis [PCoA]), were also analyzed using QIIME2 software. Additionally, differences in the gut microbiota between the two groups were further analyzed using R software (version 3.3.1), and important phyla/genera in the groups were identified using linear discriminant analysis Effect Size (LEfSe) in QIIME2 software.

### Statistical analysis

Data were expressed as mean ± standard deviation (SD), and SPSS 19.0 was used for statistical analysis. If the data were normally distributed, comparisons between the two groups were performed using the t-test, whereas, if not normally distributed, the Wilcoxon rank-sum test was performed. Count data were reported as ratios or constitutive ratios and examined using the chi-square test or Fisher’s exact test. *p* < 0.05 was considered statistically significance. Logistic regression analysis was used to determine the effects of various influencing factors on premature rupture of membranes and weight gain during pregnancy.

## Results

### General clinical data statistics

In this study, we firstly collected 102 cases, and based on the inclusion and exclusion criteria, eight cases were excluded, including 1 case with twins, 2 cases with breech out first, 3 cases with history of diabetes, and 2 cases where exercise was not recommended by obstetricians. Therefore, 94 cases were adopted for grouping, as well as 52 cases and 42 cases were used as L and M groups, respectively, based on their exercise levels. Additionally, during the experiments, there were four cases dropped out, of which one case in L group did not give birth in our hospital, one case in L group withdrew midway, as well as one case in M group did not give birth, and one case in M group failed to collect the samples. Finally, a total of 90 pregnant women (*n* = 50 for L group and *n* = 40 for M group) were included, 46 of whom were from Zhejiang, China, with a weight gain of 3.2 to 21 kg (mean ± SD: 10.7 ± 3.5) during pregnancy. Among the participants, 28 had dystocia live births, while 62 had natural live births. Furthermore, the M group consisted of 40 women, with a weight gain of 3.2 to 15 kg (mean ± SD: 11.7 ± 3.97) during pregnancy, and 11 cases of dystocia live births. At the conclusion of the study, significant differences were observed between the M and L groups in terms of membrane rupture (*p* = 0.014), K + levels (*p* = 0.036), total cholesterol levels (*p* = 0.038), and gestational weight gain (*p* = 0.001) (Table 1).

**Table 1:** Comparison of general information between the two groups.

	Indexes	Total	M	L	*p*
Birth process	Dystocia live baby	28 (31.11%)	11 (39.29%)	17 (60.71%)	0.508
	Normal childbirth	62 (68.89%)	29 (46.77%)	33 (53.23%)	
ROM	AROM	17 (18.89%)	5 (29.41%)	12 (70.59%)	**0.014**
	PROM	21 (23.33%)	15 (71.43%)	6 (28.57%)	
	SROM	52 (57.78%)	20 (38.46%)	32 (61.54%)	
	HCY	4.3 ± 0.91	4.2 ± 0.93	4.5 ± 0.89	0.22
	K^+^	4 ± 0.27	4 ± 0.3	3.9 ± 0.21	**0.036**
	Fructosamine	1.5 ± 0.12	1.5 ± 0.1	1.5 ± 0.14	0.7
	GLU	4.9 ± 0.7	4.8 ± 0.59	5 ± 0.81	0.211
	LDL-C	3.2 ± 0.55	3.3 ± 0.59	3 ± 0.47	0.058
	HDL-C	2.1 ± 0.35	2.1 ± 0.35	2 ± 0.35	0.097
	TG	2.3 ± 0.74	2.2 ± 0.7	2.4 ± 0.8	0.322
	TC	6.4 ± 0.93	6.6 ± 1.01	6.2 ± 0.77	**0.038**
	UA	264.1 ± 48.04	259.7 ± 47.86	269.6 ± 48.31	0.334
	SCR	50.8 ± 8.36	50.5 ± 8.77	51.1 ± 7.91	0.706
	AST	19.1 ± 5.43	18.1 ± 4.04	20.3 ± 6.63	0.069
	ALT	17.4 ± 10.31	16.4 ± 9.29	18.6 ± 11.47	0.336
	ALB	35.5 ± 2.15	35.4 ± 2.07	35.6 ± 2.27	0.767
	TBIL	7 ± 2.15	6.9 ± 2.24	7.2 ± 2.04	0.442
	Hb	128.2 ± 12.12	127.9 ± 10.8	128.7 ± 13.73	0.773
	TSH	1.7 ± 0.68	1.8 ± 0.74	1.6 ± 0.58	0.123
	Basic DBP	67.9 ± 8.13	67.5 ± 8.55	68.4 ± 7.66	0.627
	Basic SBP	108.5 ± 9.83	109.4 ± 9.71	107.3 ± 9.99	0.334
	postpartum blood loss	290.7 ± 105.03	298 ± 110.18	281.5 ± 98.83	0.457
	Newborn birth length	50 ± 0.42	50.1 ± 0.47	49.9 ± 0.35	0.122
	Neonatal birth weight	3326.2 ± 455.52	3353.6 ± 479.43	3,292 ± 427.28	0.522
	weight	66.9 ± 9.31	68.3 ± 10.7	65.2 ± 6.95	0.102
	DBP	73.3 ± 9.18	74 ± 9.82	72.4 ± 8.36	0.421
	SBP	118.9 ± 10.59	118.5 ± 10.36	119.4 ± 10.98	0.683
	AF	444 ± 144.47	426.6 ± 133.14	465.8 ± 156.48	0.212
	GWG	10.7 ± 3.54	11.7 ± 3.97	9.5 ± 2.43	**0.001**

M, the participants exercised 200 min a week; L, the subjects in the L group were received 150 min of aerobic exercise per week. ROM, rupture of membranes; AROM, artificial rupture of membranes (amniotomy); PROM, premature rupture of membranes; SROM, spontaneous rupture of membranes; HCY, homocysteine; GLU, glucose; LDL-C, low density lipoprotein-cholesterol; HDL-C, high density lipoprotein-cholesterol; TG, triglyceride; TC, total cholesterol; UA, uric acid; SCR, serum creatinine; AST, aspartate amino transferase; ALT, alanine aminotransferase; ALB, albumin; TBIL, total bilirubin; Hb, hemoglobin; TSH, thyroid stimulating hormone; SBP, systolic pressure; DBP, diastolic blood pressure; AF, amniotic fluid; GWG, gestational weight gain. Blod values mean *p* < 0.05.

Logistic regression analysis was used to study the effects of various influencing factors on premature rupture of membranes and weight gain during pregnancy. In the logistic regression analysis of membrane rupture, spontaneous rupture of membranes served as the control group, while artificial and premature rupture of membranes constituted the experimental group. Basic diastolic blood pressure (DBP) and alanine aminotransferase (ALT) levels were significantly correlated with the mode of membrane rupture (*p* = 0.023 for DBP, and *p* = 0.022 for ALT, Table 2). In the logistic regression analysis of weight gain during pregnancy, a weight gain of 11.5 to 16 kg served as control. Additionally, it was found that different exercise levels during pregnancy had no significant effect on the mode of membrane rupture (*p* = 0.260) or weight gain (*p* = 0.117) in pregnant women between the L and M groups (Tables 2, 3).

**Table 2:** Logistic regression analysis of premature rupture of membranes.

Logistic regression	Indexes	OR value (95%CI)	*p*
Univariate	GWG	0.9 (0.79–1.02)	0.115
	weight	0.97 (0.92–1.02)	0.259
	Basic SBP	1.04 (1–1.09)	0.065
	Basic DBP	1.07 (1.01–1.13)	**0.023**
	TSH	0.82 (0.42–1.52)	0.534
	FT	0.94 (0.65–1.32)	0.717
	Hb	1.01 (0.98–1.05)	0.459
	ALB	0.99 (0.81–1.21)	0.943
	ALT	1.06 (1.01–1.13)	**0.022**
	AST	1.04 (0.96–1.13)	0.383
	SCR	0.99 (0.95–1.05)	0.830
	UA	1 (0.99–1.01)	0.960
	TC	1.3 (0.82–2.1)	0.272
	TG	1.2 (0.68–2.15)	0.525
	HDL-C	1.1 (0.33–3.69)	0.872
	LDL-C	1.51 (0.7–3.4)	0.303
	GLU	0.96 (0.51–1.77)	0.908
	Fructosamine	0.49 (0.01–17.37)	0.695
	K^+^	0.23 (0.03–1.26)	0.116
	HCY	0.74 (0.45–1.18)	0.214
	AIDP	1.78 (0.76–4.19)	0.187
Multivariate	Basic DBP	1.08 (1.02–1.15)	**0.011**
	ALT	1.07 (1.02–1.14)	**0.013**

GWG, gestational weight gain; SBP, systolic pressure; DBP, diastolic blood pressure; HCY, homocysteine; GLU, glucose; LDL-C, low density lipoprotein-cholesterol; HDL-C, high density lipoprotein-cholesterol; TG, triglyceride; TC, total cholesterol; UA, uric acid; SCR, serum creatinine; AST, aspartate amino transferase; ALT, alanine aminotransferase; ALB, albumin; TBIL, total bilirubin; Hb, hemoglobin; TSH, thyroid stimulating hormone; AF, amniotic fluid; FT, free thyroxine; AIDP, activity intensity during pregnancy; CI, confidence interval. Blod values mean *p* < 0.05.

**Table 3:** Univariate logistic regression analysis of weight gain during pregnancy.

Indexes	OR value (95% CI)	*p*
ROM	2.24 (0.56–9.52)	0.260
weight	0.96 (0.91–1.01)	0.117
postpartum blood loss	1 (1–1.01)	0.708
Basic SBP	0.97 (0.93–1.02)	0.269
Basic DBP	0.98 (0.93–1.04)	0.559
TSH	0.64 (0.32–1.25)	0.194
FT	1.22 (0.82–1.94)	0.366
Hb	0.98 (0.94–1.02)	0.37
ALB	1.16 (0.93–1.45)	0.208
ALT	0.98 (0.94–1.02)	0.334
AST	0.99 (0.91–1.08)	0.749
SCR	1.01 (0.95–1.06)	0.831
UA	1 (0.99–1.01)	0.82
TC	0.7 (0.41–1.15)	0.170
TG	1.32 (0.7–2.65)	0.413
HDL-C	0.35 (0.09–1.31)	0.127
LDL-C	0.72 (0.3–1.68)	0.453
GLU	1.13 (0.59–2.45)	0.734
Fructosamine	10.41 (0.19–824.95)	0.272
K^+^	5.38 (0.78–51.07)	0.117
HCY	0.98 (0.59–1.65)	0.944
AIDP	1.62 (0.64–4.33)	0.322

ROM, rupture of membranes; SBP, systolic pressure; DBP, diastolic blood pressure; HCY, homocysteine; GLU, glucose; LDL-C, low density lipoprotein-cholesterol; HDL-C, high density lipoprotein-cholesterol; TG, triglyceride; TC, total cholesterol; UA, uric acid; SCR, serum creatinine; AST, aspartate amino transferase; ALT, alanine aminotransferase; ALB, albumin; TBIL, total bilirubin; Hb, hemoglobin; TSH, thyroid stimulating hormone; FT, free thyroxine; AIDP, activity intensity during pregnancy.

### Effects of different exercise levels on the overall structure of gut microbiota in GDM

**Figure 1:**
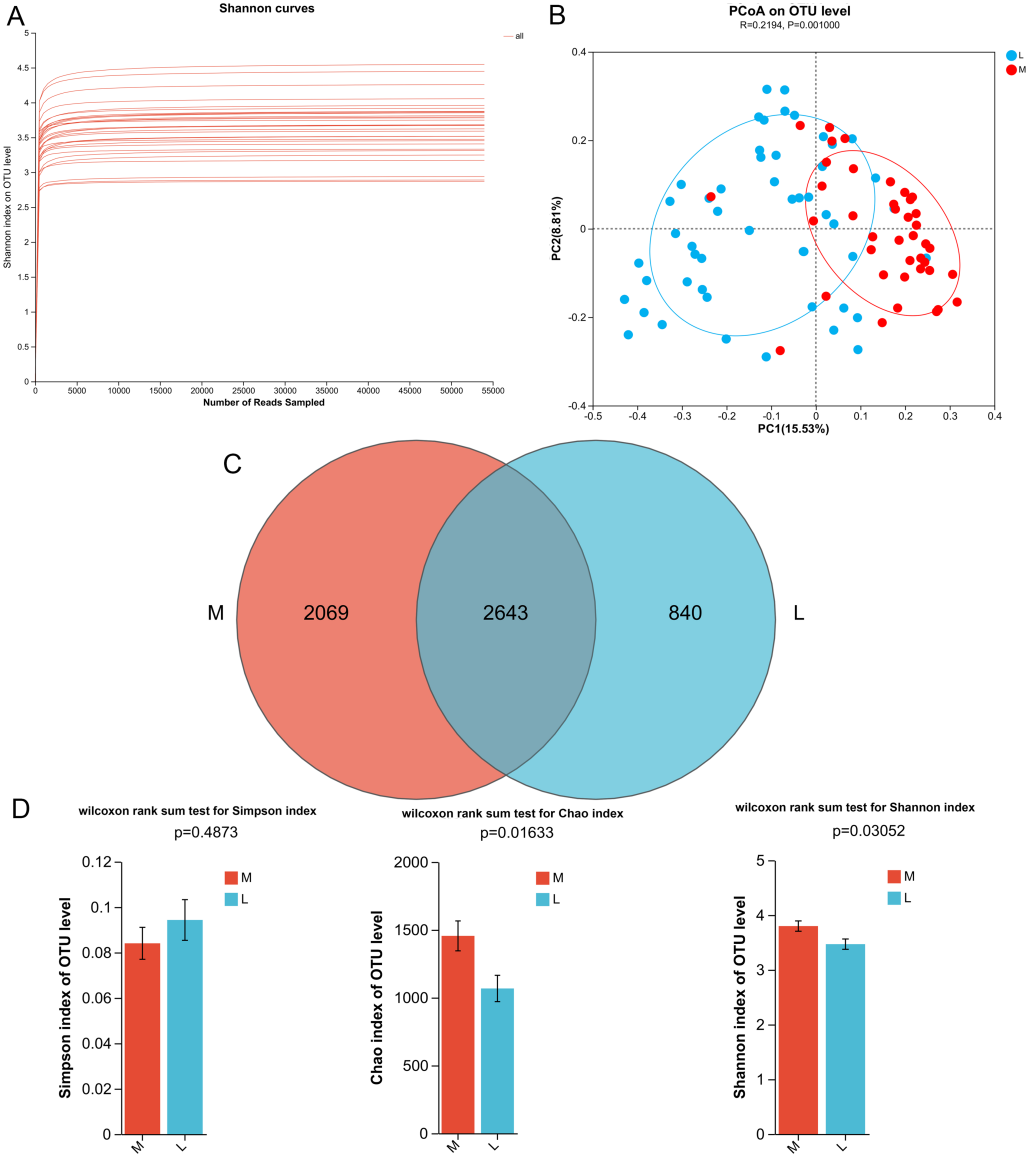
Effects of different exercise levels on the overall structure of gut microbiota in gestational diabetes mellitus (GDM). **(A)** Rank-abundance curves of all the samples; **(B)** Beta diversity analysis using pincipal coordinate analysis (PCoA) based on the Bray Curtis distance; **(C)** Venn diagram of the identified gut microbiota (OTUs) in the M and L groups. **(D)** Alpha diversity analyses based on the Simpson, Chao, and Shannon indices. M: participants who exercised 200 min per week; L: participants who received 150 min of aerobic exercise per week.

To better understand the effect of different exercise levels on the gut microbiota in GDM, fecal samples were collected for 16S rRNA gene sequencing. To verify whether the sequencing depth was sufficient to cover all OTU in the species, rank-abundance curves were plotted. As shown in Figure 1A, with an increasing number of samples, the rank-abundance curves gradually flattened, and the inflection point of the curve changes for all samples was less than 5,000, which was far below the average sequence count per sample in the experiment. These results indicated that the sample size was reasonable, and the sequencing depth was sufficient to reflect the species composition of the gut microbiota and capture most of its diversity. The PCoA results showed that the samples in each group clustered together, with significant clustering observed in the M and L groups (*p* = 0.001, Figure 1B), suggesting a significant difference in the diversity of gut microbiota between the M and L groups, which could be used for further analysis.

After 16S rRNA gene sequencing, 4,712 and 3,483 OTUs were identified in the M and L groups, respectively, with 2,643 OTUs shared between the two groups (Figure 1C). Subsequently, we calculated the α-diversity indices for the different groups, including Simpson, Chao, and Shannon indices. The Chao index reflects species richness; a higher value indicates greater species richness. The Shannon and Simpson indices were used to evaluate species diversity, where a higher Shannon index and a lower Simpson index indicate higher diversity. There was no significant difference in the Simpson index between the M and L groups (*p* = 0.4873; Figure 1D). However, compared to the L group, the Chao and Shannon indices were significantly higher in the M group (*p* = 0.01633 for Chao index, and *p* = 0.03052 for Shannon index, Figure 1D). Taken together, these findings suggest that a higher exercise level (200 min/week) can enhance the biodiversity of the gut microbiota in GDM compared to a lower exercise level (150 min/week).

### Effects of different exercise levels on the specific gut microbiota in GDM from the phylum level

**Figure 2:**
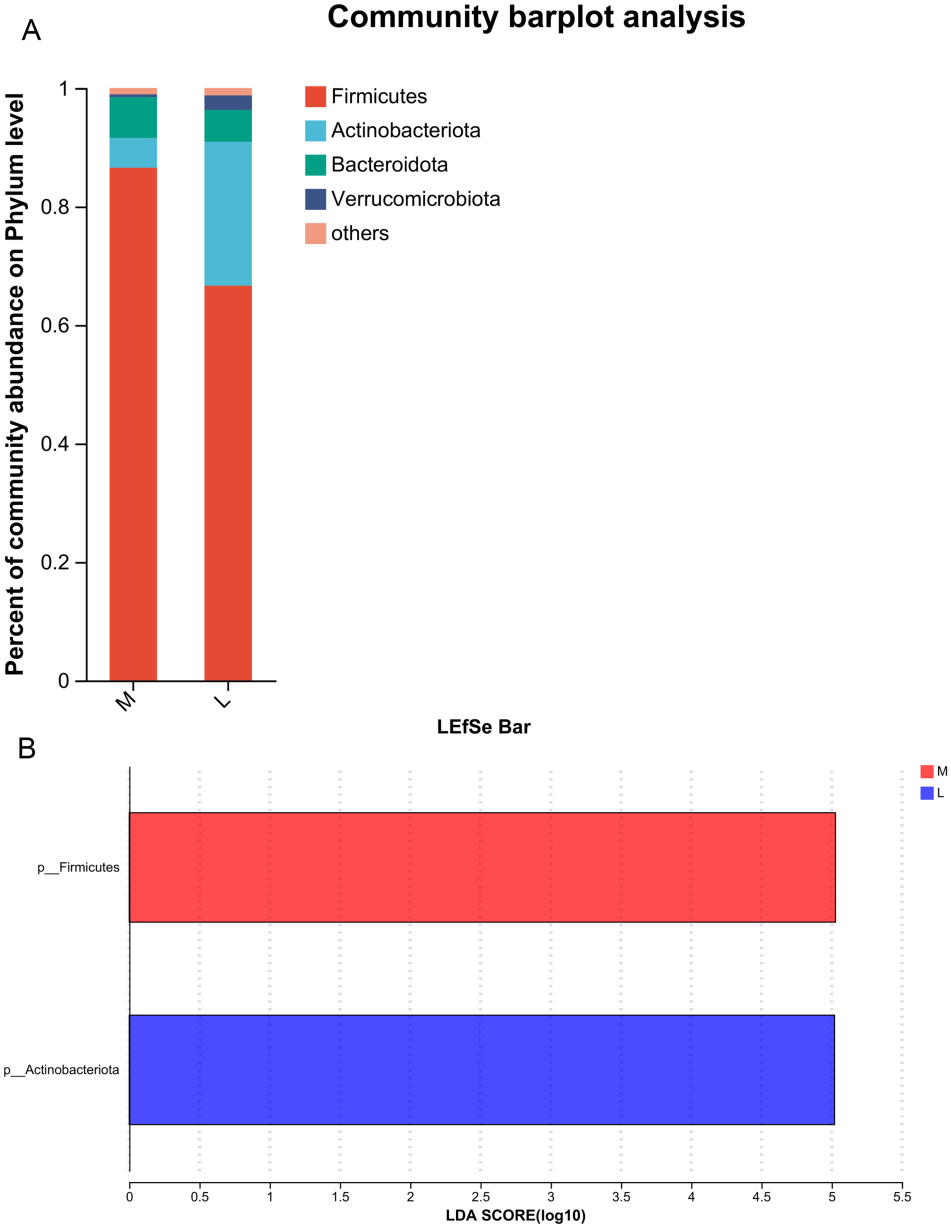
Effects of different exercise levels on the specific gut microbiota in GDM from the phylum level. **(A)** The dominant phyla of gut microbiota in the M and L groups; **(B)** Differences in the specific gut microbiota between groups using Linear discriminant analysis Effect Size (LEfSe) analysis at the phylum level. M: participants who exercised 200 min per week; L: participants who received 150 min of aerobic exercise per week.

After analyzing the diversity of the gut microbiota across the different groups, we further investigated the effects of different exercise levels on specific gut microbiota in GDM at both the phylum and genus levels. At the phylum level, the dominant phyla were *Firmicutes*, *Actinobacteriota*, *Bacteroidota*, and *Verrucomicrobiota* (Figure 2A). Among these, the relative abundances of *Firmicutes* and *Bacteroidota* were higher in the M group than in the L group, while the relative abundances of *Actinobacteriota* and *Verrucomicrobiota* were lower in the M group compared to the L group (Figure 2A). LEfSe analysis at the phylum level showed that *Firmicutes* abundance was significantly increased in the M group (87%) compared to the L group (64%, LDA score > 5), whereas *Actinobacteriota* abundance was evidently decreased in the M group (5%) relative to the L group (24%, LDA score > 5, Figure 2B). These results suggest that *Firmicutes* and *Actinobacteriota* may serve as biomarker phyla for distinguishing GDM patients with higher exercise time from those with lower exercise time.

### Effects of different exercise levels on the specific gut microbiota in GDM from the genus level

**Figure 3:**
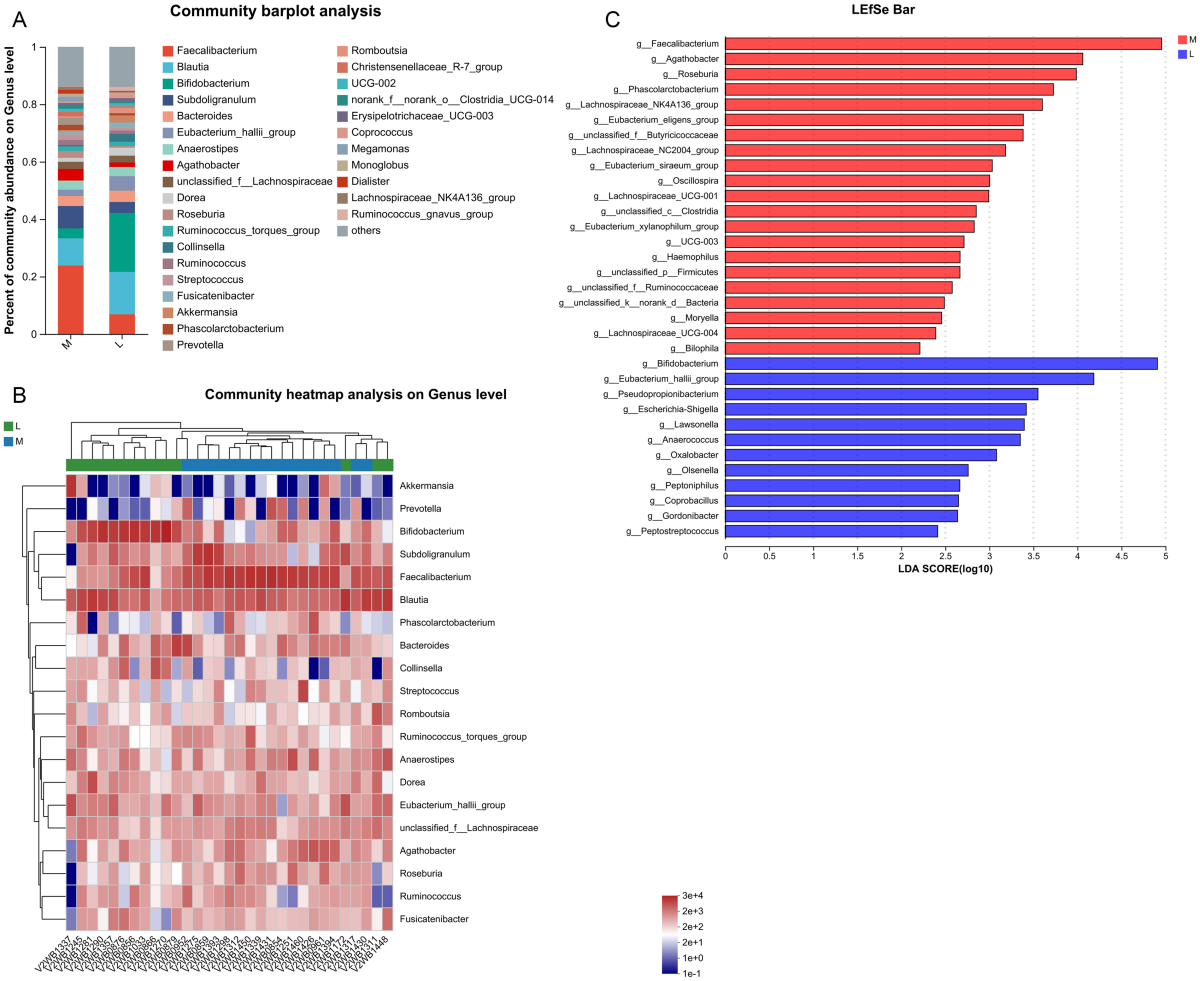
Effects of different exercise levels on the specific gut microbiota in GDM at the genus level. **(A)** Distribution of top30 dominant genera of gut microbiota in the different groups; **(B)** Clustering heatmap of the top20 genera of gut microbiota in the M and L groups; **(C)** Differences in the specific gut microbiota between the M and L groups using LEfSe analysis at the phylum level. M: participants who exercised 200 min per week; L: participants who received 150 min of aerobic exercise per week.

At the genus level, top30 bacterial genera were analyzed, including *Faecalibacterium*, *Blautia*, *Bifidobacterium*, *Subdoligranulum*, *Bacteroides*, *Agathobacter*, *Dorea*, *Roseburia*, *Collinsella*, *Akkermansia*, *Prevotella*, and *Dialister* (Figure 3A). Compared with the L group, the relative abundances of *Faecalibacterium* and *Subdoligranulum* in the M group increased from 7 to 24% and from 4 to 8%, respectively. In contrast, the relative abundances of *Blautia* and *Bifidobacterium* declined in the M group compared to the L group, from 15 to 9% and from 21 to 3%, respectively. Next, clustering heatmap analysis was performed on the top20 genera in the samples between the two groups (Figure 3B). LEfSe analysis at the genus level found that the abundances of *Faecalibacterium*, *Agathobacter*, *Roseburia*, *Phascolarctobacterium*, *Osillospira*, *Haemophilus*, *Moryella*, and *Biolphila* were significantly higher in the M group (LDA score > 2), while the abundances of *Bifidobacterium*, *Pseudopropionibacterium*, *Lawsonella*, *Anaerococcus*, *Oxalobacter*, *Olsenella*, *Peptoniphilus*, *Coprobacillus*, *Gordonibacter*, and *Peptostreptococcus* were remarkedly higher in the L group (LDA score > 2, Figure 3C). These results indicate that the aforementioned gut microbiota, such as *Faecalibacterium*, *Agathobacter*, *Roseburia*, *Osillospira*, *Bifidobacterium*, and *Coprobacillus*, are key genera for GDM patients with moderate-intensity exercise (200 min/week).

## Discussion

GDM is a common complication of pregnancy, and its incidence has increased worldwide in recent years (Sweeting et al., 2022). Large-scale epidemiological studies have found that the global incidence of GDM among pregnant women aged 20–49 years is as high as 14%, resulting in a substantial medical and economic burden (Juan and Yang, 2020; Lende and Rijhsinghani, 2020). GDM significantly threatens the lives and health of women by increasing the risk of hypertensive disorders during pregnancy, perineal trauma, and type 2 diabetes (Plows et al., 2018). Additionally, maternal glucose intolerance can lead to increase fetal glucose intake, which raises the incidence of large babies for gestational age or macrosomia, neonatal hypoglycemia, and the risk of metabolic syndrome in offspring during childhood or later in life (Catalano and Shankar, 2017). Therefore, exploring new targets to prevent or mitigate the occurrence and development of GDM is of great economic and social significance.

Lifestyle changes are essential for managing GDM, and the first-line treatment being medical nutritional therapy combined with weight management and exercise (Rasmussen et al., 2020). It has been found that in 70–85% of women diagnosed with GDM, lifestyle changes alone are sufficient to control blood sugar levels (A.D. Association, 2017). A prospective randomized clinical trial conducted at Peking University First Hospital in China showed that beginning supervised stationary cycling for at least 30 min, three times a week, in early pregnancy, could reduce the risk of GDM in overweight and obese pregnant women by 45.8% (Wang et al., 2016). Ai-Sadi et al. reported that both continuous and intermittent aerobic exercise programs can help reduce anxiety and depression in individuals with hypertension (Ai-Sadi et al., 2022), indicating that exercise therapy may be beneficial for certain conditions. A previous study showed that moderate-intensity physical exercise for at least 30 min daily, or 150 min weekly, could have a beneficial effect on glucose and insulin levels, contributing to better blood sugar control (Rasmussen et al., 2020). Our study found that different exercise levels significantly affected membrane rupture, K + levels, total cholesterol, and gestational weight gain in GDM patients, but had no significant correlation with the mode of membrane rupture and weight gain in pregnant women. These results may be due to the small sample size and minimal differences in exercise intensity. An earlier study demonstrated that blood flow restriction strength exercises reduced blood glucose levels in women with diabetes (Wagner et al., 2023). Another randomized controlled trial showed that a supervised physical exercise program (moderate aerobic exercise) started early and maintained throughout pregnancy could reduce the risk of excessive maternal weight gain and GDM (Barakat et al., 2019). Harrison et al. illustrated that moderate-intensity aerobic or resistance exercise at least three times a week can safely help control postprandial blood sugar levels and other blood sugar control measures in women diagnosed with GDM (Harrison et al., 2016). Additionally, exercise could improve blood glucose and lipid levels, and increase insulin resistance sensitivity in GDM. Taken together, it can be inferred that appropriately increasing exercise time with moderate intensity may improve pregnancy outcomes for patients with GDM as well as the growth and development of their newborns.

The pathogenesis of GDM has not been fully elucidated. The current view is that GDM involves two defects: insulin secretion and insulin action. However, the gut microbiota, an important component of the intestinal mucosal barrier, plays an important role in regulating various physiological activities and the occurrence and development of various diseases (Qi et al., 2021). With the continuous advancement of research on gut microbiota, researchers have gradually realized that the occurrence and development of GDM are closely related to intestinal microecology (Hu et al., 2023). It has been reported that gut microbiota participates in the metabolism of lipids, carbohydrates and other substances in the host, and affects the host immune regulation as a key stimulant of the immune system (Takiishi et al., 2017). The imbalance of gut microbiota, induced by changes in its abundance and diversity, can contribute to maternal inflammatory responses and IR during pregnancy (Song et al., 2022), and IR is considered the root cause of GDM (Alejandro et al., 2020). In the current study, we found that the *α*-diversity of gut microbiota in GDM patients after an intervention of 200 min/week of exercise was higher than that observed after 150 min/week of exercise. Kuang et al. demonstrated that the α-diversity of gut microbiota was significantly reduced, and the contents of *Parobacteria* and *Klebsiella* were higher in GDM patients, while the abundance of *Bifidobacterium* and *Eubacterium* was higher in healthy pregnant women, indicating that GDM progression is related to an imbalance in gut microbiota (Kuang et al., 2017). Another study observed that exercise could increase microbial biodiversity and the representation of taxa with beneficial metabolic functions, as well as enhance intestinal metabolites, indicating that exercise is a possible regulator of gut microbiome composition (Ticinesi et al., 2019). Therefore, we speculated that moderate-intensity exercise, with different duration times, may affect the α-diversity of gut microbiota in GDM, and that longer exercise duration may be more beneficial for diversity, thereby having positive effects on GDM.

In addition, we analyzed the specific gut microbiota induced by different exercise levels at the phylum and genus levels. At the phylum level, the dominant phyla were *Firmicutes*, *Actinobacteriota*, *Bacteroidota*, and *Verrucomicrobiota*. Compared with the L group, the abundance of *Firmicutes* was significantly increased, while *Actinobacteriota* abundance was evidently decreased in the M group. At the genus level, the top30 bacterial genera were identified. LEfSe analysis revealed that moderate-intensity exercise increased the levels of *Faecalibacterium*, *Agathobacter*, *Roseburia*, and *Osillospira*, while decreasing the abundance of *Bifidobacterium* and *Coprobacillus*. Ionescu et al. showed that, compared with healthy patients, those with GDM had an increase in *Firmicutes*, a decrease in *Bacteroidota* and *Actinobacteriota*, or no difference in microbiota (Ionescu et al., 2022). This trend may persist into the postpartum period or affect the fetus (Ionescu et al., 2022). Another study analyzed the gut microbiota of GDM patients at 3–16 months postpartum through 16S rRNA gene sequencing and found that *Prevotellaceae* abundance was increased, whereas *Firmicutes* abundance was reduced in postpartum GDM patients (Fugmann et al., 2015). These results suggest that the abundance of *Firmicutes* and *Actinobacteriota* may be closely associated with GDM progression. Zhang et al. found that the levels of *Agathobacter* and *Roseburia* were positively correlated with the estimated glomerular filtration rate and negatively associated with serum creatinine and microalbuminuria (Zhang et al., 2023). *Agathobacter* may be the most promising biomarkers of gut bacteria to distinguish the different stages of diabetic kidney disease. *Osillospira* is widely found in the gut of both animals and humans, and its abundance is strongly associated with obesity, thinness, and human health (Yang et al., 2021). *Bifidobacterium* is a common probiotic that positively correlates with glucagon-like peptide-1 in GDM (Liang et al., 2022). A previous study showed that the levels of *Bifidobacterium*, *Lactobacillu*s and *Bacteroides* were reduced in GDM, accompanied by an imbalance in key inflammatory cytokines such as IL-6, TNF-*α*, and hs-CRP (Chen et al., 2022). Moreover, the release and activity of inflammatory cytokines may regulate insulin sensitivity and, in turn, aggravate the inflammatory response and IR (de Mendonça et al., 2022). Another study reported that 24 weeks of exercise increased the levels of *Anaerostipes*, *Bifidobacterium*, and *Osillospira*, while decreasing the levels of *Prevotella* and *Oribacterium* in older populations (Erlandson et al., 2021). *Coprobacillus* was been found to play an important role in type 2 diabetes through various metabolic pathways, and the abundance of *Bifidobacterium longum* was negatively correlated with follow-up blood glucose levels (Wang et al., 2021). Based on our findings, we speculate that longer durations of moderate-intensity exercise (200 min/week) may help maintain the balance of the gut microbiota by regulating the abundance of *Firmicutes* (*Agathobacter*, *Roseburia*, *Osillospira*, and *Coprobacillus*) and *Actinobacteriota* (*Bifidobacterium*), thus promoting intestinal health and improving the prognosis of GDM.

Furthermore, *Faecalibacterium*, a major butyric acid-producing bacterium, accounts for 1–6% of the gut microbiota and has been suggested to be associated with IR (Leylabadlo et al., 2020). Moran-Ramos et al. showed that the abundance of *Faecalibacterium prausnitzii* is negatively correlated with the homeostasis model assessment IR index (*p* = 0.018) (Moran-Ramos et al., 2021). Similarly, Maioli et al. reported a strong relationship between *Faecalibacterium prausnitzii* and IR (Maioli et al., 2021). The main mechanism underlying the beneficial effects of *Faecalibacterium* is likely its ability to produce butyrate (Martín et al., 2023). It is worth noting that impaired intestinal barrier function is a key factor in the occurrence and development of insulin resistance. Reduced intestinal mucosal barrier function can lead to increased levels of bacterial lipopolysaccharide in the blood, which then activate the TLR pathway, triggering immune responses and damaging pancreatic *β*-cells (Matheus et al., 2017). Butyrate is the primary nutrient for the regeneration and repair of intestinal epithelial tissues. Supplementing butyrate-producing bacteria may actively repair the intestinal mucosal barrier, metabolize and consume glucose, reduce glucose absorption in the gastrointestinal tract, protect pancreatic β-cells, and lower blood glucose and liver glycogen levels (Khani et al., 2012). In addition, *Faecalibacterium* secretes a 15 kDa microbial anti-inflammatory molecule that can inhibit the NF-κB pathway in intestinal epithelial cells (Quévrain et al., 2016). This molecule can restore intestinal barrier function in diabetic patients by regulating the tight junction pathway and the expression of tight junction protein 1 (Xu et al., 2020). It has been reported that a decline in *Faecalibacterium* abundance may promote the development of type 2 diabetes (Thingholm et al., 2019) and cardiovascular disease (Tsai et al., 2021). Taken together, we speculate that increased maternal exercise of moderate intensity may improve the clinical symptoms of GDM patients and reduce the risk of complications in offspring by increasing the abundance of *Faecalibacterium*.

However, this study has some limitations. First, due to the small sample size and short intervention period, it is recommended that future research be conducted with a larger cohort and over a longer intervention period to better clarify the relationships among exercise dose, gut microbiota, and health effects. Additionally, the specific roles and potential mechanisms of the identified gut microbiota and their predicted biological pathways in women should be further investigation.

## Conclusion

16S rRNA gene sequencing was used to investigate the relationship between maternal exercise level and gut microbiota in pregnant women with GDM. It was found that significant differences existed in the composition and structure of the gut microbiota among patients with GDM with different exercise levels. Specifically, a higher exercise time with moderate intensity (200 min/week) may positively impact GDM and its offspring by increasing the biodiversity of gut microbiota and maintaining the balance of bacterial populations (e.g., *Firmicutes*: *Agathobacter*, *Roseburia*, *Osillospira*, and *Coprobacillus*; *Actinobacteriota*: *Bifidobacterium*). Additionally, *Faecalibacterium* may be the most promising biomarker of intestinal bacteria in patients with GDM undergoing longer exercise periods with moderate intensity. Our findings provide new evidence of the regulatory effect of exercise on gut microbiota in patients with GDM, offering novel insights for the precise guidance of exercise therapy and the promotion and implementation of exercise prescriptions, with *Faecalibacterium* as a potential biological indicator of GDM exercise intervention.

## Data Availability

The 16S rRNA sequencing data have been deposited in the Genome Sequence Archive (GSA) (https://ngdc.cncb.ac.cn/gsa/) at accession number CRA010697.

## Footnotes

^1^ https://ngdc.cncb.ac.cn/gsa/
